# Modelling Carbon Nanotubes-Based Mediatorless Biosensor

**DOI:** 10.3390/s120709146

**Published:** 2012-07-03

**Authors:** Romas Baronas, Juozas Kulys, Karolis Petrauskas, Julija Razumiene

**Affiliations:** 1 Faculty of Mathematics and Informatics, Vilnius University, Naugarduko 24, LT-03225 Vilnius, Lithuania; E-Mail: romas.baronas@mif.vu.lt; 2 Institute of Biochemistry, Vilnius University, Mokslininku 12, LT-08662 Vilnius, Lithuania; E-Mails: juozas.kulys@bchi.vu.lt (J.K.); julija.razumiene@bchi.vu.lt (J.R.)

**Keywords:** modelling, simulation, reaction-diffusion, biosensor, mediatorless, SWCNT

## Abstract

This paper presents a mathematical model of carbon nanotubes-based mediatorless biosensor. The developed model is based on nonlinear non-stationary reaction-diffusion equations. The model involves four layers (compartments): a layer of enzyme solution entrapped on a terylene membrane, a layer of the single walled carbon nanotubes deposited on a perforated membrane, and an outer diffusion layer. The biosensor response and sensitivity are investigated by changing the model parameters with a special emphasis on the mediatorless transfer of the electrons in the layer of the enzyme-loaded carbon nanotubes. The numerical simulation at transient and steady state conditions was carried out using the finite difference technique. The mathematical model and the numerical solution were validated by experimental data. The obtained agreement between the simulation results and the experimental data was admissible at different concentrations of the substrate.

## 1. Introduction

Biosensors are devices mainly used to measure concentrations of substances (analytes) [1,2]. Main parts composing a biosensor are biologically active material, usually an enzyme, selectively detecting target species and a transducer converting a chemical detection event into an electrical signal. The electrical signal is then amplified, processed and presented to the end-user. Biosensors are highly sensitive and reliable devices and therefore are widely used in medicine, food industry, environment monitoring and for drug detection [3,4]. According to “Global Industry Analysts Inc.”, the global market for the biosensors was $8.2 billion in 2009 and was expected to grow by 6.3% annually [5].

Amperometric biosensors measure the changes in the current on the working electrode due to the oxidation or reduction of the products of biochemical reactions [6]. Mediated biosensors as a kind of amperometric biosensors require the participation of redox molecules in signal transduction. The mediated biosensors are often constructed with the enzymes that can donate electrons to electrochemically active artificial electron acceptors [7]. A more advanced method is to have no mediator and to ensure the direct electron transfer between the enzyme and the electrode [8]. The mediatorless biosensors are the so-called third generation biosensors and are successors for the mediated biosensors [8–10]. The mediatorless biosensors are advantageous over the mediator-based biosensors because of the simplicity in their application and modelling. The biosensors with the direct electron transfer have higher selectivity and are less prone to the interfering reactions [11–13]. One of the main obstacles in the development of the mediatorless biosensors is that only few enzymes can support the direct electron transfer [14].

Since the discovery of carbon nanotubes [15] they have been used in various applications. Due to their unique structural and electric properties, carbon nanotubes (CNT) have been used to build highly sensitive biosensors [16–18]. Recently, an innovative approach in the design of biosensors with the CNT mesh deposited on the polycarbonate perforated membrane has been proposed [19], and a novel family of mediated biosensors acceptable for detection of wide range of carbohydrates have been designed [20]. The use of CNTs opens an opportunity for the direct electron transfer between the enzyme and the electrode [21]. The application of glucose dehydrogenase and CNTs permitted to build mediator free glucose biosensor [21].

The development and optimization of new biosensors require a high number of experiments. Mathematical modelling is rather often used in order to decrease the number of physical experiments by replacing them with mathematical simulations [22,23]. Starting from 1970s, mathematical modelling has been proved as an effective tool to study and optimize analytical characteristics of actual biosensors [24–26]. A comprehensive review on modelling of the amperometric biosensors has been presented by Schulmeister [27] and more recently by Baronas *et al.* [28].

A CNT-based biosensor was mathematically modelled by Lyons [29,30]. The one-dimensional-in-space boundary value problem describing the transport and the kinetics of the substrate and the mediator in the two compartment domain was solved analytically, assuming steady state conditions. Practical biosensors are usually built covering them with outer porous or perforated membranes [3,6,19]. The mathematical model for the mediated biosensor with the CNT electrode deposited on the perforated membrane was proposed recently [31].

This paper presents a mathematical model of the mediatorless amperometric biosensor based on the enzyme-loaded CNT electrode deposited on the outer perforated membrane. The proposed model describes the unmediated operation of the biosensor having the geometrical structure similar to that of the already modelled mediated biosensor [19,31]. The model is based on nonlinear non-stationary reaction-diffusion equations. The new model is described in a one-dimensional-in-space domain and comprises four layers (compartments): a layer of enzyme solution entrapped on a terylene membrane, a layer of the single walled carbon nanotubes, a perforated membrane and an outer diffusion layer. The numerical simulation at transient and steady state conditions was carried out using the finite difference technique [28,32,33]. The mathematical model and the numerical solution were validated by experimental data. The obtained agreement between the simulation results and the experimental data was admissible at different substrate concentrations. The biosensor response and sensitivity were numerically investigated by changing the model parameters with a special emphasis on the mediatorless transfer of the electrons in the layer of the enzyme-loaded CNTs.

## 2. Principal Structure of the Biosensor

The investigated biosensor has the layered structure and is composed of different materials and sizes according to [19]. The active surface of the biosensor is built by binding the mesh of the single wall carbon nanotubes to the perforated membrane. Some of the nanotubes are sinked into the holes of the membrane during the preparation procedure. Then CNTs were pre-oxidized enzymatically using laccase from *Basidiomycete Lac*. After this procedure, the layer of CNT was throughly washed with distilled water up to total clearing of laccase and covered by the layer of the enzyme. The changeable enzyme layer of proposed CNT-based biosensor was designed by immobilization of soluble type of pyrroloquinoline quinone dependent glucose dehydrogenase from *Acinetobacter calcoaceticus* L.M.D. 79.41 to the semi-permeable membrane of terylene.

**Figure 1.**
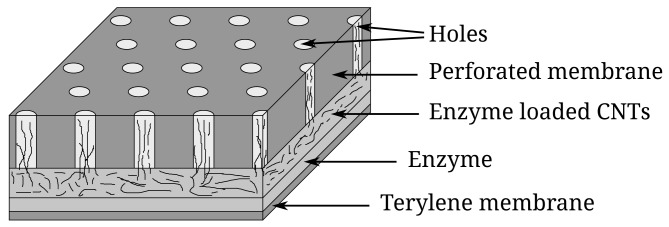
Principal structure of the active surface of the biosensor. The figure is not to scale.

All electrochemical experiments were performed using a conventional three-electrode system containing a planar CNT electrode as a working electrode, a platinum wire as a counter electrode and an Ag/AgCl in saturated KCl as a reference electrode. The default buffer was 0.05 M acetate buffer (pH 6.0) containing 1 mM Ca^2+^. Steady state currents of the biosensors were recorded at 0.4 V using a polarographic analyzer “PARSTAT 2273” (Princeton Applied Research, USA). Principal structure of the considered biosensor is shown in Figure 1.

Enzymatic reaction is employed in the biosensor to selectively detect the substrate (S) in the target analyte. The enzymatic reaction takes place in the regions of the biosensor filled with the enzyme,

(1) $${\text{E}}_{\text{ox}}+\text{S}\overset{k_{1}}{\to }{\text{E}}_{\text{red}}+\text{P},$$

where *k*_1_ is a constant of the enzymatic reaction rate. In the reaction, the substrate S reacts with the oxidized form of the enzyme (E_ox_) and reduces it (E_red_) producing the product P. The latter is considered as not impacting the processes in the biosensor and therefore is omitted in the following model.

The output current of the biosensor is generated due to the direct enzyme oxidation taking place in the layer of the carbon nanotubes,

(2) $${\text{E}}_{\text{red}}\overset{k_{2}}{\to }{\text{E}}_{\text{ox}}+n_{e}{\text{e}}^{-},$$

where *k*_2_ is a constant of the electrochemical reaction rate and *n*_e_ is the number of electrons released in one reaction event. The enzyme E_red_ is re-oxidized in the Reaction (2) releasing electrons that are collected by the CNT electrode.

## 3. Mathematical Model

The mathematical model for the biosensor is formulated as a system of non-linear reaction-diffusion equations with the corresponding initial and boundary conditions. The model is formulated in the one-dimensional space by applying the homogenization process to the perforated membrane and the mesh of carbon nanotubes [34,35]. A line perpendicular to the biosensor surface is considered as the domain of the model with the zero point at the surface of the terylene membrane. The model involves four layers (Ω*_i_*) with different properties,

(3) $$\begin{array}{cccc} \Omega_{i}=\left(x_{i-1},x_{i}\right), & x_{0}=0,\;x_{i-1}+d_{i}, & \Gamma_{0}=\left\{0\right\},\;\Gamma_{i}=\left\{x_{i}\right\}, & i=1,2,3,4, \end{array}$$

where *d*_1_ is the thickness of the enzyme layer existing between the CNTs and the terylene membrane, *d*_2_ is the thickness of the CNT layer, *d*_3_ is the thickness of the perforated membrane and *d*_4_ stands for diffusion layer forming on the outer surface of the perforated membrane.

### 3.1. Governing Equations

The enzyme in the oxidized form participates in the enzymatic Reaction (1) in both enzyme-loaded regions, Ω_1_ and Ω_2_. Additionally, the enzyme that is properly conjoined with the active sides of the CNTs is also involved in the electrochemical Reaction (2) and is re-oxidized. Due to the procedure of the mediatorless biosensor preparation, it was assumed that only a part of the whole enzyme has direct contact with the electro-active sides of the CNTs, and only that part of the enzyme is involved in the electrochemical Reaction (2) [19,20]. The dynamics of the concentration of the oxidized enzyme can be mathematically described as follows (*t* > 0):

(4) $$\begin{array}{cc} \frac{\partial E_{\text{ox},i}}{\partial t}=-k_{1}E_{\text{ox},i}S_{i}, & \;x\in \Omega_{i},i=1,2, \end{array}$$

(5) $$\begin{array}{cc} \frac{\partial E_{\text{e},\text{ox},2}}{\partial t}=-k_{1}E_{\text{e},\text{ox},2}S_{2}+k_{2}E_{\text{e},\text{red},2}, & x\in \Omega_{2}, \end{array}$$

where *x* stands for the space, *t* is time, *S_i_* = *S_i_*(*x, t*) is the substrate concentration in the *i*^th^ layer Ω*_i_*, *E*_ox,*i*_ = *E*_ox,*i*_(*x, t*) is the concentration of the oxidized enzyme that is not involved in the Reaction (2). *E*_e,ox,2_ = *E*_e,ox,2_(*x, t*) and *E*_e,red,2_ = *E*_e,red,2_(*x, t*) are the concentrations of the enzyme involved in the Reaction (2) in the oxidized and the reduced forms, respectively.

The dynamics of the concentration of the reduced enzyme is practically opposite to that of the oxidized enzyme. E_red_ is produced in the enzymatic Reaction (1) in both the enzyme-loaded layers. The electrochemically active part of the reduced enzyme (*E*_e,red,2_) is additionally re-oxidized in the electrochemical Reaction (2) (*t* > 0),

(6) $$\begin{array}{cc} \frac{\partial E_{\text{red},i}}{\partial t}=k_{1}E_{\text{ox},i}S_{i}, & \;x\in \Omega_{i},i=1,2, \end{array}$$

(7) $$\begin{array}{cc} \frac{\partial E_{\text{e},\text{red},2}}{\partial t}=-k_{1}E_{\text{e},\text{ox},2}S_{2}-k_{2}E_{\text{e},\text{red},2}, & x\in \Omega_{2}, \end{array}$$

where *E*_red,*i*_ = *E*_red,*i*_(*x, t*) is the concentration of the reduced enzyme that is not involved in the Reaction (2).

The molecules of both forms of the enzyme, E_ox_ and E_red_, are considered as immobilized, and therefore there are no diffusion terms in the corresponding equations.

The substrate S diffuses from the bulk through the holes of the perforated membrane to inner layers of the biosensor. The substrate also participates in the enzymatic Reaction (1) in the enzyme-loaded layers (Ω_1_ and Ω_2_). The dynamics of the substrate concentration is described by the following reaction-diffusion equations:

(8) $$\frac{\partial S_{i}}{\partial t}=\{\begin{array}{lll} D_{{\text{S}}_{i}}\frac{\partial^{2}S_{i}}{\partial x^{2}}-k_{1}E_{\text{ox},i}S_{i}, & \text{for}\;i=1, & \\ D_{{\text{S}}_{i}}\frac{\partial^{2}S_{i}}{\partial x^{2}}-k_{1}E_{\text{ox},i}S_{i}--k_{1}E_{\text{e},\text{ox},i}S_{i}, & \text{for}\;i=2,\;, & x\in \Omega_{i},t>0,\\ D_{{\text{S}}_{i}}\frac{\partial^{2}S_{i}}{\partial x^{2}}, & \text{for}\;i=3,4, & \end{array}$$

where *D*_S_1__ and *D*_S_4__ are the diffusion coefficients of the substrate S in the enzyme and the diffusion layers, respectively. The coefficients *D*_S_2__ and *D*_S_3__ stand for the effective diffusivity of the substrate in the CNT layer and the perforated membrane, respectively.

### 3.2. Boundary Conditions

Assuming well stirred buffer solution leads to the constant thickness of the diffusion layer as well as the constant concentration above that layer,

(9) $$\begin{array}{cc} {S_{4}|}_{\Gamma_{4}}=S_{0}, & t>0, \end{array}$$

where *S*_0_ is the concentration of the substrate in the bulk solution.

The terylene membrane is placed between the enzyme and the insulating layer of the biosensor and plays a role of an insulating film immobilizing the enzyme. Assuming low volume of the membrane and the insulating layer behind it, the non-leakage condition is used for the substrate on the surface of the terylene membrane (Γ_0_),

(10) $$\begin{array}{cc} {\frac{\partial S_{1}}{\partial x}|}_{\Gamma_{0}}=0, & t>0. \end{array}$$

On the boundaries between adjacent regions (Ω*_i_* and Ω*_i_*_+1_, *i* = 1,2,3), the merge conditions are defined for the substrate:

(11) $$\begin{array}{cccc} {D_{{\text{S}}_{i}}\frac{\partial S_{i}}{\partial x}|}_{\Gamma_{i}}={D_{{\text{S}}_{i+1}}\frac{\partial S_{i+1}}{\partial x}|}_{\Gamma_{i}}, & {S_{i}|}_{\Gamma_{i}}={S_{i+1}|}_{\Gamma_{i}}, & i=1,2,3, & t>0. \end{array}$$

The diffusion layer Ω_4_ (*x*_3_ < *x* < *x*_3_+*d*_4_) may be treated as the Nernst diffusion layer [33]. According to the Nernst approach a layer of thickness *d*_4_ remains unchanged with time.

### 3.3. Initial Conditions

The modelled experiment starts (*t* = 0) when the substrate is poured into the buffer solution. At this time, the substrate is absent in the entire biosensor and the diffusion layer, except only the outer boundary Γ_4_, where the substrate concentration is considered equal to that in the bulk solution,

(12) $$\begin{array}{ccccc} S_{i}=0, & x\in {\bar{\Omega }}_{i}\backslash \Gamma_{4}, & {S_{4}|}_{\Gamma_{4}}=S_{0}, & i=1,2,3,4, & t=0, \end{array}$$

where Ω̄*_i_* is the closed region corresponding to the open region Ω*_i_*.

At the beginning of the experiment all the enzyme is assumed to be in the oxidized form. The initial concentrations of the oxidized (*E*_ox,1_) and the reduced (*E*_red,1_) enzyme in the enzyme layer Ω_1_ are defined as follows:

(13) $$\begin{array}{cccc} E_{\text{ox},1}=E_{0}, & E_{\text{red},1}=0, & x\in \Omega_{1}, & t=0, \end{array}$$

where *E*_0_ is the enzyme concentration in the layer filled with the enzyme only (Ω_1_).

Due to the procedure of the mediatorless biosensor preparation, the concentration of the enzyme in the CNT layer is assumed to be lower than in the bulk [19,20]. Moreover, only a part of the enzyme is properly conjoined with the CNTs to be electrochemically active. Assuming a uniform distribution of the enzyme in the CNT layer leads to following conditions:

(14) $$\begin{array}{ccccc} E_{\text{ox},2}=\left(1-\alpha \right)\eta E_{0}, & E_{\text{e},\text{ox},2}=\alpha \eta E_{0}, & E_{\text{red},2}=0, & x\in \Omega_{2}, & t=0, \end{array}$$

where *η* (0 ≤ *η* ≤ 1) is the ratio of the enzyme concentration in the CNTs to that in the enzyme layer Ω_1_, and *α* (0 ≤ *α* ≤ 1) is the volume fraction of the electrochemically active enzyme in the CNT layer.

### 3.4. Biosensor Response and Sensitivity

The output current of the biosensor is generated due to the enzyme re-oxidation in the electrochemical Reaction (2) taking place only in the enzyme-loaded CNT electrode (region Ω_2_). It was assumed that only the enzyme molecules properly attached to the CNTs (*E*_e,red,2_) can be re-oxidized in the Reaction (2). The density *j*(*t*) of the output current is proportional to the rate of the electrochemical Reaction (2),

(15) $$j(t)=n_{\text{e}}Fk_{2}\int_{x_{1}}^{x_{2}}E_{\text{e},\text{red},2}\text{d}x,$$

where *F* is the Faraday constant.

In practice, the current generated at a steady state is often used as an output of the biosensor. Assuming that the system approaches the steady state as *t* → ∞ leads to the following definition of the density *J* of the steady state output current of the considered biosensor:

(16) $$J=\underset{t\to \infty }{\lim}j(t).$$

The sensitivity is one of the most important characteristics of the biosensor operation [1,2]. The normalized sensitivity to the substrate concentration *B_S_* was measured when investigating the impact of the model parameters on the behaviour of the biosensor,

(17) $$B_{S}(S_{0})=\frac{\text{d}\;J(S_{0})}{\text{d}\;S_{0}}\times \frac{S_{0}}{J(S_{0})}.$$

### 3.5. Effective Diffusion Coefficients

The enzyme and the diffusion layers were assumed to be homogeneous. The diffusion coefficients of the substrate S in these layers are constant,

(18) $$\begin{array}{cc} D_{{\text{S}}_{1}}=D_{\text{e}}, & D_{{\text{S}}_{4}}=D_{\text{n}}, \end{array}$$

where *D*_e_ is the diffusion coefficient of the substrate for the enzyme layer, and *D*_n_ is the corresponding coefficient for the bulk.

The CNT layer and the perforated membrane both are non-homogeneous mediums. Assuming these layers as periodic media, the volume averaging approach can be applied to estimate the effective (averaged) diffusion coefficients for these layers [34–36]. The CNT layer is considered as a composite of three compartments: the carbon nanotubes, the enzyme and the bulk. The perforated membrane is treated as a composite of the non-permeable material the membrane is built of and the bulk in the holes of the membrane together with the carbon nanotubes sunk in. Assuming relatively low volume fraction of the CNTs, the diffusion coefficients of the substrate can be expressed in terms of the volume fractions of the compounds, the diffusion coefficients in the specific compounds and the tortuosity:

(19) $$D_{{\text{S}}_{2}}=\theta_{2}\left(\eta D_{\text{e}}+\left(1-\eta \right)D_{\text{n}}\right),$$

(20) $$D_{{\text{S}}_{3}}=\theta_{3}\rho D_{\text{n}},$$

where *ρ* stands for the perforation level of the membrane as the ratio of the volume occupied by the holes to the overall volume of the membrane, *θ*_2_ and *θ*_3_ are the tortuosities describing the structural properties of the corresponding medium, < 0 < *θ*_2_, *θ*_3_ < 1. Values of the tortuosities *θ*_2_ and *θ*_3_ mainly depend on the directional anisotropy of the CNTs, particularly on the nanotubes orientation [29]. A near-unity tortuosity corresponds to a fully vertical alignment of the CNTs (Figure 1). The effect of the directional anisotropy of the nanotubes has been already investigated for the corresponding mediated biosensor with the CNT electrode deposited on the perforated membrane [31].

Similar approach to estimating the effective diffusion coefficients was applied to the two-dimensional model for the mediated biosensor based on a CNT electrode [31] and the one-dimensional model for the biosensor with the outer perforated membrane [37].

## 4. Digital Simulation

The formulated mathematical model (4)–(16) was defined as an initial boundary value problem involving non-linear reaction terms. Analytical solutions for such type of systems are known only for several special cases, therefore numerical approximation is usually employed to get solutions for wide ranges for the model parameters [27,28].

### 4.1. Simulation Technique

In this work the method of finite differences was used to solve numerically the system of the proposed model [32]. The model domain was discretized by applying the regular mesh to each space region Ω*_i_*(*i* = 1,2,3,4), and a constant step was used to discretize the time. The model equations were approximated by semi-implicit finite difference schemes. The diffusion terms were approximated by the backward differences, while the reaction terms were approximated by the forward differences [38]. The resulting system of linear tri-diagonal algebraic equations was solved effectively. The computer simulation was carried out using a software developed by the authors in C++ programming language [39].

In the numerical simulations, the steady state was assumed at the time, when the increase of the output current becomes small enough,

(21) $$T_{\text{R}}=\underset{j(t)>0}{\min}\left\{t:\frac{\text{d}\;j(t)}{\text{d}\;t}\times \frac{t}{j(t)}<\varepsilon \right\},$$

where *T*_R_ is the time when the steady state results in the output current *j*(*T_R_*), and *ε* is the decay rate of the output current to be considered in the simulations. In all the simulations presented in this paper *ε* = 0.01 was used.

The following parameter values were used as a basic configuration of the considered biosensor and were kept constant in all the experiments:

(22) $$\begin{array}{c} \begin{array}{cccc} d_{1}=10^{-7}\text{m}, & d_{2}=4\times 10^{-1}\text{m}, & d_{3}=10^{-5}\text{m}, & d_{4}=3\times 10^{-4}\text{m}, \end{array}\\ \begin{array}{cccccc} D_{\text{e}}=3\times 10^{-10}{\text{m}}^{2}{\text{s}}^{-1}, & D_{\text{n}}=2D_{\text{e}}, & \eta =0.5, & \theta_{2}=1/3, & \theta_{3}=0.5, & \rho =0.0625 \end{array}\\ \begin{array}{cc} n_{\text{e}}=2, & k_{1}=6.9\times 10^{2}{\text{m}}^{3}{\text{mol}}^{-1}{\text{s}}^{-1}. \end{array} \end{array}$$

### 4.2. Experimental Validation

**Figure 2.**
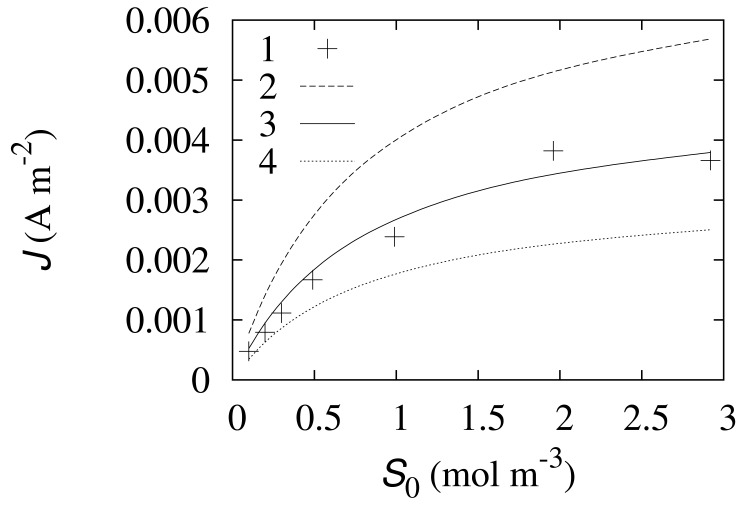
The densities (*J*) of steady state current obtained numerically (2, 3, 4) and experimentally (1). The simulations were performed at *k*_2_ = 550 s^−1^, *E*_0_ = 0.0455 mol m^−3^ and three values of the ratio *α* : 0.0075 (2), 0.005 (3) and 0.0033 (4). The other parameters are as defined in Reaction (22).

**Figure 3.**
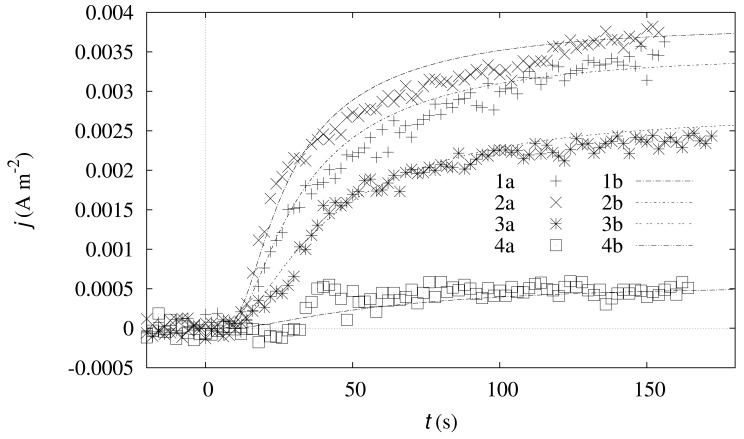
The dynamics of the density *j*(*t*) of the output current obtained experimentally (1a, 2a, 3a and 4a) and numerically (1b, 2b, 3b and 4b) at four substrate concentrations (*S*_0_): 0.099 (1a, 1b), 0.99 (2a, 2b), 1.96 (3a, 3b) and 2.92 mol m^−3^ (4a, 4b). The simulation was performed at *α* = 0.005, while the other parameters are the same as in Figure (2). The horizontal and vertical dotted lines stand for the zero axes.

The mathematical Model (4)–(16) and the corresponding numerical solution were validated by experimental data. The experimental data along with the corresponding simulated responses of the biosensor are shown in Figures 2 and 3.

It is difficult to experimentally measure the concentration of the enzyme involved in the electrochemical Reaction (2). The volume fraction *α* of the electrochemically active enzyme highly depends on the properties of carbon nanotubes and the technology of the electrode preparation [20]. The rate constant *k*_2_ of the electrochemical Reaction (2) is also very specific for the developed CNT electrode. Multiple numerical simulations were performed in order to estimate the values of the *α*- and *k*_2_-parameters. These two parameters were fitted by minimizing the relative error between the simulated and experimental responses. A satisfactory match was obtained at *α* = 0.005 and k_2_ = 550 s^−1^, when only 0.5% of the enzyme is assumed as participating in the electrochemical Reaction (2) in the CNT layer. The steady state responses of the simulated as well as physical experiments are shown in Figure 2.

As it can be observed in Figure 2, the responses obtained in numerical experiments using *α* = 0.005 are close to the experimental data. At that value of *α*, the relative error of the simulated steady state responses is less than 10%, except two concentrations *S*_0_ of the substrate: *S*_0_ = 0.199 and 0.299 mol m^−3^. At low substrate concentrations the relative error reaches almost 20%. Taking into account relatively low biosensor currents and possible measuring errors in the physical experiments, the error of the simulated responses can be considered admissible. The fitted value 550 s^−1^ of the electrochemical reaction rate constant *k*_2_ suitably matches with reported values of apparent heterogeneous electron transfer rate constants [9].

Figure 2 also shows how sensitive is the biosensor response to the *α*-parameter. The steady state current directly depends on the *α*. Increasing the volume fraction *α* of the electrochemically active enzyme increases the biosensor current and prolongs the linear part of the biosensor calibration curve.

The proposed model was also validated at the transient conditions. The simulation was carried out using the same biosensor configuration as in the simulations shown in Figure 2. The dynamics of the simulated density *j*(*t*) of the biosensor current along with the corresponding experimental data are shown in Figure 3. The oscillations in the experimental data shown in Figure 3 are caused by the measurement errors. Due to relatively low output currents, the errors are relatively high. Taking into account the measurement errors, the simulated responses can be assumed adequate to that observed in the physical experiments.

When modelling the corresponding mediated biosensor, no similar parameter was used [31]. The response of the mediated biosensor based on an enzyme-loaded CNTs was modelled assuming whole enzyme involved in the corresponding mediated electrochemical reaction. Multiple numerical simulations of the response of the mediatorless biosensor showed that involving the whole enzyme into the electrochemical Reaction (2) leads to the steady-state currents being in about two order greater than the experimental ones. Because of this, the volume fraction *α* of the electrochemically active enzyme is of crucial importance when modelling the mediatorless biosensor based on enzyme loaded CNT electrode.

## 5. Results and Discussion

The behaviour of the response of the mediatorless biosensor was investigated by performing numerical simulations based on the developed mathematical Model (8)–(16). In order to investigate the influence of the model parameters related to the direct electron transfer on the biosensor current as well as on the sensitivity, the simulation was performed at wide ranges of the values of the following two parameters: the electrochemical reaction rate constant *k*_2_ and the concentration *E*_0_ of the enzyme.

### 5.1. Impact of Enzyme Concentration

The proper conjunction of the enzyme with the active sites of the CNTs is essential for the mediatorless biosensor operation. The proposed model of the biosensor assumes the proportional dependence between the total concentration of the enzyme and the concentration of the enzyme capable to participate in the electrochemical Reaction (2). To investigate the impact of the enzyme concentration *E*_0_ on the behaviour of the biosensor action, the biosensor responses were simulated in wide ranges of the enzyme (*E*_0_) as well as the substrate (*S*_0_) concentrations.

**Figure 4.**
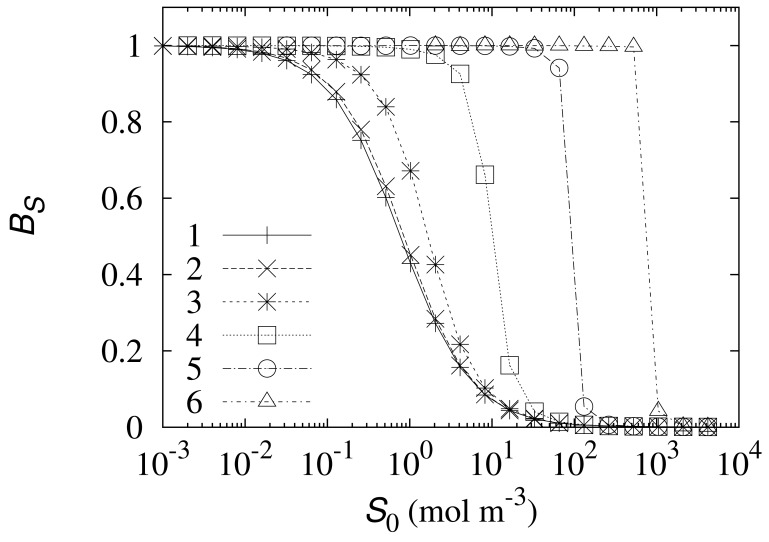
The dimensionless biosensor sensitivity *B_S_* versus the substrate concentration *S*_0_ at *k*_2_ = 550 s^−1^, *α* = 0.005 and six enzyme concentrations *E*_0_: 0.0455 (1), 0.455 (2), 4.55 (3), 45.5 (4), 455 (5) and 4, 550mol m^−3^ (6). Other parameters are as defined in Reaction (22).

The biosensor current varies in orders of magnitude as the concentrations of these species change, therefore the dimensionless sensitivity *B_S_* was considered instead of the density *J* of the steady state current. The simulation results are provided in Figure 4.

As one can see in Figure 4, an increase in the enzyme concentration *E*_0_ noticeably prolongs the linear part of the biosensor calibration curve, ensuring qualitative determination of higher substrate concentrations. The linear part of the biosensor calibration curve corresponds to *B_S_* ≈ 1. At the high enzyme concentrations (*E*_0_ > 40mol m^−3^) the length of the linear part of the calibration curve is practically proportional to the enzyme concentration *E*_0_, while at the low enzyme concentrations (*E*_0_ < 4mol m^−3^) the sensitivity of the biosensor loosely depends on the concentration *E*_0_. The especially drastic change in the sensitivity appears at the moderate values of *E*_0_. Figure 4 shows that a tenfold increase of the enzyme concentration from 45.5 (curve 4) to 455mol m^−3^ (curve 5) leads to an approximately 50 times longer linear part of the biosensor calibration curve.

The noticeable change in the behaviour of the biosensor sensitivity (Figure 4) at the moderate enzyme concentrations could be explained by the transition from the kinetic-limited to the diffusion-controlled mode of the biosensor action [26–28]. The diffusion module *σ*^2^ essentially compares the rate of the enzyme Reaction (*k*_1_*αE*_0_) with the mass transport through the enzyme-loaded layer $\left(D_{{\text{S}}_{2}}/d_{2}^{2}\right)$, $\sigma^{2}=k_{1}\alpha E_{0}d_{2}^{2}/D_{{\text{S}}_{2}}$. The biosensor response is known to be under diffusion control when the diffusion module *σ*^2^ ≫ 1. In the very opposite case, when *σ*^2^ ≪ 1, the enzyme kinetics predominates in the response. The diffusion module *σ*^2^ approaches one at the concentration *αE*_0_ of the electrochemically active enzyme approximately equal to 0.75 mol m^−3^. This value of *αE*_0_ favorably matches with the enzyme concentrations at which the behaviour of the biosensor sensitivity distinctly changes (see Figure 4).

### 5.2. Impact of Electrochemical Reaction Rate

Mathematical models for amperometric biosensors are usually formulated assuming electrochemical reactions to be extremely fast when all the available reagent is immediately consumed [27,28]. In modelling the mediatorless biosensor, it was assumed that only a small fraction of the enzyme participates in the electrochemical reaction taking place in the CNT electrode. Because of the relatively spare sites, where the direct electron transfer is possible, the electrochemical Reaction (2) cannot be assumed very fast. The appropriate value 550 s^−1^ of the *k*_2_-parameter was fitted by minimizing the relative error between the simulated and experimental responses.

**Figure 5.**
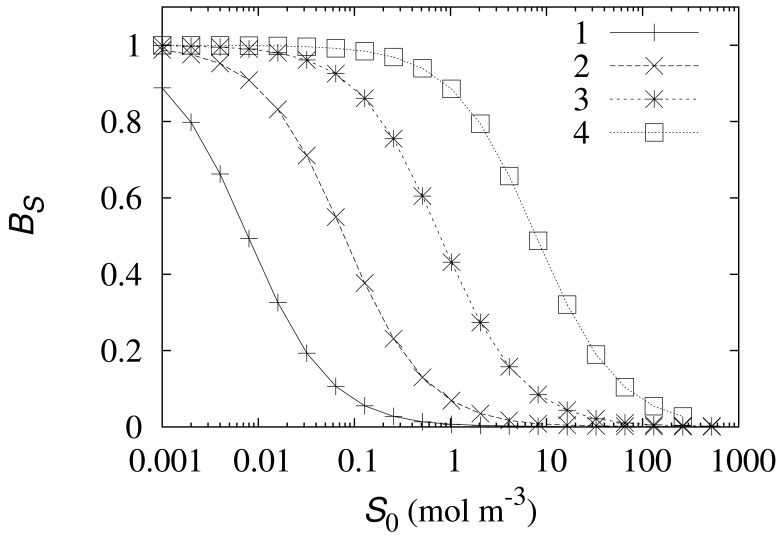
The dimensionless biosensor sensitivity *B_S_* versus the substrate concentration *S*_0_ at *α* = 0.005, *E*_0_ = 0.0455 mol m^−3^ and four values of the electrochemical reaction rate constant *k*_2_: 5.5 (1), 55 (2), 550 (3) and 5,500 s^−1^ (4). Other parameters are as defined in Reaction (22).

In order to show the impact of the rate constant *k*_2_ of the electrochemical Reaction (2) on the biosensor response, the response was numerically simulated and the corresponding dimensionless biosensor sensitivity *B_S_* was calculated at different values of the *k*_2_-parameter by changing the substrate concentrations *S*_0_ in a wide range. The simulation results are depicted in Figure 5.

As one can see in Figure 5 an increase in the electrochemical reaction rate constant *k*_2_ proportionally shifts the curve representing the sensitivity *B_S_* to the right. Thus an increase in the coefficient *k*_2_ proportionally prolongs the linear part of the biosensor calibration curve. The shape of the curve remains unchanged for all *k*_2_ used in the investigation.

## 6. Conclusions

The developed mathematical Model (8)–(16) can be used to simulate the mediatorless biosensor based on the enzyme-loaded CNT electrode. The model was validated by the experimental data at both the steady state (Figure 2) and the transient conditions (Figure 3). The relative error of the simulated steady state responses was less than 10% for all the concentrations except the concentrations generating very low responses.

The numerical simulation showed that only a fraction of the enzyme participates in the direct electron transfer at the nanoscale (Figure 2). A good match with the experimental data was obtained when only 0.5% of the enzyme loaded in the CNT electrode was considered electrochemically active. The overall rate of the electrochemical Reaction (2) for the mediatorless biosensor was determined to be relatively low. Therefore, the electrochemical reaction rate constant *k*_2_ was introduced into the mathematical Model (8)–(16).

The sensitivity of the mediatorless biosensor based on the enzyme-loaded CNT electrode can be noticeably increased and the linear part of the calibration curve can be prolonged by increasing the volume fraction *α* of the electrochemically active enzyme (Figure 2).

The biosensor sensitivity can be also significantly increased by increasing the enzyme concentration. The linear part of the calibration curve is longer when the biosensor acts in the diffusion-limiting mode rather than in the enzyme reaction-controlled mode (Figure 4).

To prove the conclusions made, physical experiments are running using biosensors acceptable for detection of a wide range of carbohydrates with a tunable selectivity.
